# Strategies for premedication and G-CSF application in sacituzumab govitecan treatment of patients with triple-negative breast cancer: multicenter insights

**DOI:** 10.1007/s00520-025-09918-4

**Published:** 2025-09-28

**Authors:** Małgorzata Pieniążek, Anna Polakiewicz-Gilowska, Marcin Kubeczko, Manuela Las-Jankowska, Renata Pacholczak-Madej, Paulina Kilian-Van Miegem, Marek Ziobro, Aleksandra Łacko, Michał Jarząb, Miroslawa Püsküllüoğlu

**Affiliations:** 1 Department of Oncology, Wrocław Medical University, Plac Hirszfelda 12, Wrocław, 53-413 Poland; 2 Lower Silesian Comprehensive Cancer Center, Plac Hirszfelda 12, Wrocław, 53-413 Poland; 3 Breast Cancer Unit, Gliwice Branch, Maria Skłodowska-Curie National Research Institute of Oncology, Gliwice, 44-102 Poland; 4 Surgical Oncology, Ludwik Rydygier Collegium Medicum in Bydgoszcz, Nicolaus Copernicus University in Torun, Oncology Centre, Bydgoszcz, 85-067 Poland; 5 Department of Clinical Oncology, Oncology Center-Prof Franciszek Lukaszczyk Memorial Hospital, Bydgoszcz, 85-796 Poland; 6 Department of Anatomy, Jagiellonian University Medical College, Świętej Anny 12, Krakow, 31-008 Poland; 7 Department of Gynecological Oncology, Maria Sklodowska-Curie National Research Institute of Oncology, Garncarska Street 11, Krakow Branch, 31-115 Poland; 8 Department of Chemotherapy, The District Hospital, Szpitalna Street 22, Sucha Beskidzka, 34-200 Poland; 9 Oncology Center Prof. F. Łukaszczyk in Bydgoszcz, Chemotherapy Outpatient Clinic, Bydgoszcz, 85-796 Poland; 10 Department of Clinical Oncology, Kraków Branch, Maria Sklodowska-Curie National Research Institute of Oncology, Kraków, Poland

**Keywords:** Sacituzumab govitecan, TNBC, Premedication, G-CSF, neutropenia prevention, Adverse events, Supportive care

## Abstract

**Background:**

Sacituzumab govitecan (SG) is approved for advanced triple-negative breast cancer (TNBC) in the second-line setting and beyond, offering improved survival compared to chemotherapy. Adverse events (AEs) commonly include myelosuppression, gastrointestinal disturbances, and hepatic toxicity. Effective premedication and prophylaxis are critical for AE management. This study assessed premedication protocols for SG-treated TNBC patients.

**Materials and methods:**

A retrospective cohort study across five oncology centers in Poland analyzed premedication regimens for patients completing SG treatment by October 2024. Premedication evaluated included acetaminophen, corticosteroids, antihistamines, 5-HT3 and NK1 receptor antagonists, and granulocyte colony-stimulating factor (G-CSF) when needed. AEs were assessed using National Cancer Institute—Common Terminology Criteria for Adverse Events, version 5.0.

**Results:**

Among 67 patients with TNBC who finished their treatment with SG, the mean age at SG initiation was 51.5 ± 12.4 years. Premedication in the first cycle included corticosteroids and 5-HT3 receptor antagonists in 66 (99%) and 65 (97%) patients, respectively; acetaminophen in 57 patients (85%), H1 blockers in 53 patients (79%), and H2 blockers in 52 patients (78%). G-CSF was used as primary prophylaxis in 27 patients (40%) and required in 53 patients (79%) during treatment. Atropine premedication was needed in 4 patients (6%) for grade 3 diarrhea as secondary prophylaxis.

**Conclusions:**

Most patients received standardized premedication regimens, but significant variability was observed in G-CSF use for neutropenia prophylaxis. Only 6% of patients required atropine as diarrhea premedication. Practices regarding the use of G-CSF varied across centers, reflecting evolving SmPC guidance and individual risk-based approaches to neutropenia management.

**Supplementary Information:**

The online version contains supplementary material available at 10.1007/s00520-025-09918-4.

## Introduction

Triple-negative breast cancer (TNBC) is one of the most aggressive forms of breast cancer (BC). It lacks the expression of estrogen and progesterone receptors (ER and PR) and shows negative human epidermal growth factor receptor 2 (HER2) status [1, 2]. Treatment options for this subtype are limited, making sacituzumab govitecan (SG) a significant advancement for patients who have exhausted first-line therapies [1–3].

SG is an antibody drug conjugate (ADC) that consists of a monoclonal antibody targeting trophoblast cell surface antigen 2 (TROP-2), a hydrolyzable linker, and SN-38, a cytotoxic agent derived from irinotecan, with a drug-to-antibody ratio (DAR) of ~ 7.6. SN-38 acts as a topoisomerase I inhibitor, inducing double-strand DNA damage and apoptosis [4–6]. SG is administered intravenously, binds TROP-2 on tumor cells, and undergoes receptor-mediated endocytosis. In the lysosome, the linker is cleaved, releasing SN-38 intracellularly [4, 5, 7]. A distinctive feature of SG is its “bystander effect,” where released SN-38 diffuses into neighboring tumor cells, enhancing its efficacy in heterogeneous tumors [6, 7].

The most common adverse events (AEs) of SG include neutropenia, anemia, diarrhea, nausea, fatigue, and alopecia [3, 8–12]. SG is metabolized in the liver. Its active component, SN-38, is inactivated by uridine diphosphate-glucuronosyltransferase 1A1 (UGT1A1). Homozygosity for the *UGT1A1*28 allele increases toxicity risk [6]. Compared to conventional irinotecan, SG delivers significantly higher tumor concentrations of SN-38, improving therapeutic efficacy while reducing off-target toxicities [4–6]. Despite its well-established toxicity profile, formal guidelines for supportive care interventions, particularly prophylactic antiemetics and granulocyte colony-stimulating factor (G-CSF), remain undefined. This gap between known adverse effects and the absence of standardized supportive measures highlights the importance of real-world evidence. Neutropenia, including febrile neutropenia, remains one of the most clinically significant adverse effects associated with sacituzumab govitecan, often necessitating dose modifications, treatment delays, or supportive care with G-CSF. The updated Summary of Product Characteristics (SmPC) (June 2025) has broadened the formal guidance on both primary and secondary prophylaxis, emphasizing the need for individualized risk assessment.

The recent Delphi consensus emphasized the need for such observational data to guide clinical management of SG-related toxicities, especially in the absence of standardized guidelines [6].

## Materials and methods

### Patient selection

The study included patients diagnosed with inoperable or metastatic TNBC.

TNBC was defined as ER and PR negativity (< 1% nuclear staining) and HER2 status of 0 or 1 + by immunohistochemistry (IHC). In cases with HER2 IHC score of 2 +, confirmation of HER2 negativity by fluorescence in situ hybridization (FISH) was required [13, 14].

Patients were treated within the national reimbursement program for SG, which requires histologically confirmed TNBC, measurable disease per Response Evaluation Criteria in Solid Tumors version 1.1, and good performance status (0–1). Additional eligibility criteria include absence of symptomatic or progressing central nervous system metastases after local therapy, absence of uncontrolled comorbidities, and adequate organ function. Full inclusion and exclusion criteria for the reimbursement access program in Poland are included in Supplementary Materials 1 [15]. There were no restrictions regarding the age or sex of the patients.

### Study design

The study was designed as a multicenter, retrospective cohort study, involving data collection from five oncology units across Poland. Patients were required (1) to have completed at least one full cycle of SG (day 1 and 8) and (2) to complete the treatment with SG regardless of the reason (tumor progression, toxicity, death, or patient decision). Both retrospective and prospective data were included, focusing on patients who had completed SG therapy by October 1, 2024.

The study adhered to the principles outlined in the Good Reporting of Outcomes in Real-world Evidence Studies (GROW) guidelines. The methodology comprehensively covered essential components of real-world evidence reporting, including study design, population characteristics, data collection methods, approaches to managing missing data, and safety evaluations [16].

This study aims to evaluate premedication practices for sacituzumab govitecan (SG) therapy across multiple oncology centers in Poland, focusing on commonly used protocols, variation in clinical practice, and the use of G-CSF for neutropenia management. As a secondary objective, the study also examines treatment-related toxicity and modifications in premedication or supportive care during the course of SG therapy.

### SG dosing and schedule

The recommended dose of SG is 10 mg/kg body weight, administered as an intravenous infusion on days 1 and 8 of a 21-day treatment cycle. No patients in this cohort received treatment on a fixed biweekly schedule (e.g., days 1 and 15 every 28 days). Any delays or deviations were transient and related to toxicity or individual clinical circumstances.

The infusion was administered over a period of 3 h during the first administration and was shortened to 1–2 h in subsequent cycles if well tolerated [17]. Patients receiving SG should be monitored closely throughout the infusion and for a minimum of 30 min afterward for any signs of infusion-related reactions. Treatment continued until disease progression, death, patient’s consent withdrawal or the occurrence of unacceptable toxicity.

### Premedication protocols

As per the SmPC [17], premedication before each administration of SG is recommended to minimize the risk of infusion-related reactions and chemotherapy-induced nausea and vomiting (CINV). To prevent infusion-related reactions, patients should receive antipyretics, histamine H1 and H2 receptor antagonists, and corticosteroids (e.g., 50 mg hydrocortisone or equivalent). For CINV prophylaxis, an antiemetic regimen should be selected based on the emetogenic risk profile and may include a combination of a corticosteroid, a 5-hydroxytryptamine type 3 (5-HT3) receptor antagonist, and a neurokinin 1 (NK1) receptor antagonist.

G-CSF could be administered prophylactically based on the patient’s neutropenia risk, especially for those with a history of myelosuppression. More data regarding dosing modifications for neutropenia based on SmPC and recently updated characteristics SmPC is presented in Supplementary Materials 2 and 3. However, updated version was published after the study ended.

Atropine administration is recommended for patients who experience pronounced cholinergic symptoms, such as abdominal cramping, diarrhea, and excessive salivation. In such cases, atropine may be used to mitigate these adverse effects in subsequent treatment cycles [17].

### Data collection

Data were collected on the premedication regimens administered prior to SG treatment, including acetaminophen, corticosteroids, 5-HT3 receptor antagonists, NK1 receptor antagonist, olanzapine, H1 and H2 blockers, and G-CSF (filgrastim or pegfilgrastim) for primary or secondary prophylaxis. The analysis also captured any modifications made to the premedication protocols during subsequent treatment cycles. Additionally, data included baseline demographic and clinical characteristics such as patient age, sex, performance status, histological diagnosis, metastatic sites, *BRCA* mutation status (if applicable), PD-L1 expression (where relevant), and prior lines of systemic therapy received. Information on the number of treatment cycles completed, dose adjustments, treatment delays, and reasons for treatment discontinuation was also recorded. Adverse events were evaluated according to the National Cancer Institute Common Terminology Criteria for Adverse Events (NCI-CTCAE), version 5.0.

Some of the data, particularly eligibility criteria and key laboratory results, were collected prospectively within the framework of routine documentation required for SG reimbursement. Additional clinical variables, including details of premedication and supportive care, were extracted retrospectively by physician investigators. Premedication practices were not standardized across institutions and were determined individually by the treating physicians. For missing data, source documentation was reviewed when available; otherwise, such cases were explicitly noted.

### Statistical considerations

The statistical analysis aimed to describe premedication strategies and supportive care practices. Descriptive statistics were used to summarize baseline patient characteristics, treatment patterns, and the incidence of adverse events. Continuous variables, such as patient age, were presented as means with standard deviations or medians with interquartile ranges, as appropriate. Categorical variables, including premedication regimens, were reported as counts and percentages.

All statistical analyses were performed using validated software, with a focus on data accuracy and clarity in reporting treatment patterns and safety outcomes.

### Ethical considerations

This study was approved by the ethical committees of the Maria Sklodowska-Curie National Research Institute of Oncology in Krakow (2/2023, 18 April 2023) and Warsaw (21/2024, 22 February 2024). All procedures involving human participants complied with institutional regulations and the ethical principles of the 1964 Helsinki Declaration and its amendments. Written informed consent was obtained from all patients before initiating sacituzumab govitecan treatment under the national reimbursement program. Ethical approval confirmed that consent for retrospective data collection was not required.

## Results

### Population characteristics

Eighty-six female patients with TNBC had SG initiated within 5 oncology centers by 30 September 2024. Sixty-seven patients who had completed treatment with SG were included into analysis. At the initiation of SG therapy, their mean age was 51.5 years, accompanied by a standard deviation of 12.4 years (range: 24.6–71.2 years). The Ki-67 index had a mean of 57.3% ± 25.2% (range: 5–100%; *n* = 57). Notably, 19 (28%) of the participants were initially diagnosed with a non-TNBC subtype. At the beginning of the study, all patients presented with distant metastases. Other data regarding sociodemographic characteristic as well as previous treatment received are presented in Table 1.

**Table 1:** Patient characteristics

Parameter		Total (*N* = 67)
Disease stage at initial diagnosis	De novo metastatic	10 (15%)
	Early-stage	57 (85%)
Comorbidities*	No	39 (58%)
	Yes	28 (42%)
Menopausal status	Premenopausal	22 (33%)
	Postmenopausal	45 (67%)
Site of metastatic disease at SG initiation	Lymph nodes	54 (81%)
	Lungs	36 (54%)
	Bones	28 (42%)
	Skin/subcutaneous tissue	27 (40%)
	Liver	22 (33%)
	Malignant effusion	9 (13%)
	Brain	7 (10%)
	Other	6 (9%)

*Comorbidities requiring active management and deemed clinically significant by the treating physician

### Systemic treatment characteristics

Patients received a median of 2 treatment lines in palliative setting prior to SG initiation. 33 patients (49%) had received SG in the second line of palliative treatment, 24 (36%) after two prior lines, 4 (6%) after three lines, 4 (6%) after four lines, and one patient each (1%) after five and six lines, respectively. Majority of patients received systemic treatment in (neo)adjuvant setting as presented in Table 2. The median count of completed SG cycles was four, extending through day 8 of the fourth cycle.

**Table 2:** Characteristics of treatment received prior to SG treatment

Parameter		Total (*N* = 67)
(Neo)adjuvant systemic treatment type (*N* = 57)	Cytotoxic agents	55 (82%)
	Pembrolizumab	2 (3%)
	Hormonal therapy	10 (15%)
	Anti-HER2 agents	4 (6%)
Parameter		Total (*N* = 67)
	Cytotoxic agents	55 (82%)
(Neo)adjuvant systemic treatment type (*N* = 57)	Pembrolizumab	2 (3%)
	Hormonal therapy	10 (15%)
	Anti-HER2 agents	4 (6%)
Surgery in early-stage setting	Yes	51 (76%)
Radiotherapy in early-stage setting	Yes	43 (64%)
Palliative systemic therapy	Anthracycline-based	17 (25%)
	Taxane-based	26 (39%)
	Platinum-based	34 (51%)
	Capecitabine	20 (30%)
	Gemcitabine	17 (25%)
	Vinorelbine	10 (15%)
	Hormonal therapy	6 (9%)
	Anti-HER2 agents	0 (0%)
	Pembrolizumab	4 (6%)
	PARP inhibitors	3 (4%)
	Other agents	15 (22%)
AE grade 2 or more of any previous systemic treatment	Cardiac toxicity	1 (1%)
	Diarrhea	6 (9%)
	Vomiting	9 (13%)
	Nausea	10 (15%)
	Hypersensitivity reactions	2 (3%)
	Increased ALT/AST	16 (24%)
	Anemia	23 (34%)
	Thrombocytopenia	9 (13%)
	Neutropenia	26 (39%)
	Neutropenic fever	1 (1%)

*Patient could receive more than one agent

*AE* Adverse adverse event, *ALT/AST *alanine aminotransferase/aspartate aminotransferase, *BC *breast cancer, *HER2 *human epidermal growth factor receptor 2, *PARP *poly (ADP-ribose) polymerase

### Treatment tolerance and toxicity

The most common grade 3 or higher toxicity was neutropenia with median time of occurrence on day 1 of cycle 2 (see Table 3). The treatment was stopped due to AEs in 2 patients (3%). Seventeen patients (25%) required at least one dose reduction (in 5 cases due to neutropenia G4, in 10 cases due to neutropenia any grade). In 48 patients, the treatment was delayed due to AE (mainly, in 14 patients due to neutropenia G3, in 31 cases due to neutropenia of any type).

**Table 3:** Sacituzumab govitecan treatment safety data

Parameter	Any grade (*N* = 67)	Grade 3 or higher (*N* = 67)
Diarrhea	9 (13%)	3 (4%)
Vomiting	2 (3%)	0 (0%)
Nausea	7 (10%)	1 (1%)
Increased ALT or AST	13 (19%)	3 (4%)
Anemia	30 (45%)	8 (12%)
Platelet count decrease	10 (15%)	3 (6%)
Neutropenia	45 (67%)	33 (49%)
Neutropenic fever	2 (3%)	2 (3%)
Hypersensitivity reactions	ND	1 (2%)

*AE* adverse event, *ALT/AST* alanine aminotransferase/aspartate aminotransferase, *ND* no data

### Premedication strategies within first cycle

During the first treatment cycle, 57 patients (85%) received 1 g of intravenous acetaminophen. Steroids, primarily 8 mg of intravenous dexamethasone, were administered to 66 patients (99%), and 5-HT3 receptor antagonists, most commonly 8 mg of intravenous ondansetron, were given to 65 patients (97%). H1 blockers, such as 2 mg of intravenous clemastine, were administered to 53 patients (79%), while H2 blockers, primarily 40 mg of oral famotidine, were provided to 52 patients (78%). These data are summarized in Table 4.

**Table 4:** Premedication agents administered during the first cycle of sacituzumab govitecan treatment

Premedication type	Drug	Dose	No. of patients	% of patients
Antipyretic	Acetaminophen	1 g	57	85%
Steroid (antiemetic)	Dexamethasone	8 mg 12 mg*	65 1	97% 1%
5-HT3 receptor antagonist (antiemetic)	Ondansetron ND	8 mg ND	63 2	94% 3%
NK1 receptor antagonist (antiemetic)	Aprepitant	125 + 80 + 80 mg	1	1%
Dopamine antagonist (antiemetic)	Metoclopramide	10 mg	1	1%
Atypical antipsychotic (antiemetic)	Olanzapine	5 mg	1	1%
H1 blocker** (antiallergic)	ND	ND	53	79%
H2 blocker*** (antiallergic)	ND	ND	52	78%

*12 mg day 1, and 8 mg in subsequent days for patient that received aprepitant

*2 mg of iv clemastine as per local standards in all centers, but data for every patient were not collected

**40 mg of oral famotidine as per local standards in all centers, but data for every patient were not collected

*5-HT3* 5-hydroxytryptamine type 3; *H1* histamine type 1; *H2* histamine type 2; *ND *no data; NK1 neurokinin-1

### Premedication strategies within subsequent cycles

Three patients had acetaminophen added during subsequent cycles, while 7 patients (10%) did not receive acetaminophen throughout the entire treatment. One patient (1%) did not receive steroids at any point. Two additional patients (3%) required iv H1 blockers in later cycles, including one due to a hypersensitivity reaction, and one additional patient (1%) needed oral H2 blockers in subsequent cycles. The use of 5-HT3 antagonists increased slightly to 66 patients (99%) during subsequent cycles. Four patients (6%) required the initiation of an oral NK1 receptor antagonist (in 3 aprepitant, standard doses for 3 days; in one patient, a fixed-dose combination of netupitant 300 mg/palonosetron 0.5 mg was added; consequently, ondansetron was discontinued in this patient). Additionally, four patients required oral olanzapine (5–10 mg) and two oral metoclopramide (10 mg) in later cycles to manage persistent nausea.

### Granulocyte colony-stimulating factor use

During treatment, 53 patients (79%) received G-CSF, either as primary (27 patients, 40%) or secondary (26 patients, 39%) prophylaxis. Among those receiving primary prophylaxis, G-CSF regimens included:

- Pegfilgrastim or lipegfilgrastim after day 8 (7 patients, 10%)
- Filgrastim between days 1 and 8, followed by pegfilgrastim or lipegfilgrastim after day 8 (6 patients, 9%)
- Filgrastim only after day 8 (8 patients, 12%)
- Filgrastim both between days 1–8 and after day 8 (6 patients, 9%)

In the secondary prophylaxis group, 3 patients (4%) received pegfilgrastim or lipegfilgrastim, and 23 patients (34%) were treated with filgrastim.

Filgrastim was typically administered for a median of 4 consecutive days starting on day 2 or 3, and for 5 consecutive days starting on day 9 or 10. Pegfilgrastim or lipegfilgrastim was given as a single dose on day 9 or 10.

G-CSF was most often initiated due to a history of neutropenia or febrile neutropenia during prior treatment lines. In subsequent cycles, the leading indication for G-CSF use was grade 3 neutropenia, most commonly occurring after day 1 of cycle 2.

### Diarrhea management

Atropine was not used as a preventive measure in any patient during the first cycle. However, 4 patients (6%) required it before subsequent cycles due to the development of grade 3 diarrhea. Although symptomatic management with antidiarrheal agents was commonly applied later in treatment, detailed data on specific medications, such as loperamide, were not captured in a standardized manner and are not included in this analysis.

## Discussion

The phase 3 ASCENT trial demonstrated a significant clinical benefit of SG in heavily pretreated metastatic TNBC, improving both progression-free and overall survival compared to standard chemotherapy. However, its use is linked to AEs, including myelosuppression, gastrointestinal toxicity (diarrhea, vomiting), and liver enzyme elevations [3]. These data have been confirmed in real world studies (RWS) confirming SG efficacy [3, 8, 9, 18–20]. The considerations regarding SG premedication strategies gain further relevance in light of the recently presented ASCENT-03 and ASCENT-04 studies, which evaluated SG in the first-line setting for metastatic TNBC. Both ASCENT-03 and ASCENT-04 showed a significant PFS benefit with manageable toxicity. ASCENT-04 supported SG plus pembrolizumab in PD-L1–positive TNBC, while ASCENT-03 demonstrated efficacy in the PD-L1–negative population [21–23]. As SG is now being positioned earlier in the treatment sequence, a growing number of patients will likely be exposed to it.

There are some differences in the reporting of safety data for grade 3 or higher AEs as per CTCAE v. 5.0 in published RWSs and the ASCENT trial. These differences pertain not only to the frequency of various AEs reported but also to the methods used for reporting this data [3, 8, 9, 18–20]. Typically, the incidence of grade 3 or higher neutropenia in RWS is slightly lower compared to the 51% reported in the phase 3 ASCENT trial [3, 8, 18–20]. The incidence of febrile neutropenia reported in RWS aligns with that observed in the ASCENT trial, remaining around 6% [3, 8]. In contrast, the incidence of diarrhea was reported as 10% in the ASCENT trial [3], while RWSs showed a wide range from 4 to 19% [3, 8, 12, 18]. In our cohort, the incidence of grade 3 or higher diarrhea was 4%. Grade 3 or higher neutropenia occurred in 49% of patients, yet the rate of febrile neutropenia remained low, affecting only two patients (3%).

Effectively managing these AEs is crucial for maintaining the balance between treatment efficacy and patient health-related quality of life. Premedication protocols involving agents such as corticosteroids, acetaminophen, antihistamines, and granulocyte colony-stimulating factors (G-CSF) are typically employed to prevent or mitigate AEs. Although some recommendations may appear straightforward [24], there remains considerable variation in how premedication strategies for SG are implemented in clinical practice, particularly regarding the use of G-CSF and antiemetics. The recent Delphi consensus further underscored the lack of standardization in supportive care for patients receiving SG. Several key issues failed to reach expert agreement—even after second-round resubmissions—including the routine use of primary G-CSF prophylaxis irrespective of risk factors, the omission of G-CSF in patients with homozygous UGT1A1 *28/*28, and the appropriateness of initial dose reductions in frail or comorbid patients not represented in clinical trials [6]. The absence of consensus on these key aspects of SG administration illustrates the challenges clinicians face in routine practice and signals a clear need for prospective evidence to inform more consistent treatment strategies. The frequent need for G-CSF suggests that myelosuppression is a significant and persistent issue in this patient population, often necessitating proactive management by the second treatment cycle.

The PRIMED was an open-label, single-arm, phase II study designed to assess whether primary prophylaxis with G-CSF and loperamide could improve the tolerability of SG [25]. In this study, patients had received one or two prior chemotherapy regimens for advanced breast cancer. When primary prophylaxis was employed, the rates of any-grade neutropenia and diarrhea during the initial two treatment cycles were 28% and 34%, respectively. Severe neutropenia (grade 3 or higher) was observed in 16% of patients, and grade 3 or higher diarrhea occurred in 16%, with 4% experiencing grade 3 episodes. AEs led to dose reductions in 18% and treatment interruptions in 44% of patients, but no treatment discontinuations due to toxicity were reported.

According to the most recent NCCN guidelines, SG has been added to the list of regimens with an intermediate risk for febrile neutropenia (10–20%) [26]. In this risk category, the use of G-CSF as primary prophylaxis may be considered based on individual patient risk factors. These include prior chemotherapy or radiotherapy, persistent neutropenia, bone marrow involvement, recent surgery or open wounds, liver or renal dysfunction, age > 65 years receiving full-dose chemotherapy, and poor performance status. The presence of one risk factor is sufficient to justify consideration of primary G-CSF prophylaxis from the first treatment cycle. For patients not receiving G-CSF initially, secondary prophylaxis is recommended in cases where neutropenia results in delays to subsequent treatment cycles [26].

G-CSF administration schedules vary across guidelines and clinical protocols, particularly for regimens, where the cytotoxic agent is administered on days 1 and 8. In the PRIMED study, primary prophylaxis involved filgrastim on days 3, 4, 10, and 11 [25]. However, when this prophylactic approach was evaluated by a panel of clinical experts, it did not achieve consensus for routine use, indicating that individualized strategies remain preferred [6]. The NCCN guidelines recommend administering filgrastim until post-nadir absolute neutrophil count recovery to near-normal levels [26]. For pegfilgrastim, a minimum interval of 12 days is recommended between injection and the initiation of the subsequent chemotherapy cycle [26].

In terms of gastrointestinal toxicity, the need for atropine to manage diarrhea was limited, indicating that diarrhea associated with SG is generally well-controlled with conventional antidiarrheals, although close monitoring remains essential. In our cohort, no patient received atropine as primary prophylaxis, and only 6% required atropine prior to subsequent cycles due to the development of grade 3 diarrhea. Grade 3 or higher diarrhea was observed in 5% of patients and was well managed with standard antidiarrheal treatment. In a pooled safety analysis of 1063 patients across four studies involving various epithelial tumors, including IMMU-132-01, ASCENT, TROPiCS-02, and TROPHY-U-01, grade 3 or higher diarrhea occurred in 11% of patients, and 20% received atropine [27]. In expert opinion, routine atropine prophylaxis is not recommended, but its introduction is advised following the first episode of early-onset diarrhea or cholinergic syndrome [6].

According to the recent European Society For Medical Oncology-Multinational Association for Supportive Care in Cancer guideline update for the prevention of chemotherapy-induced nausea and vomiting, SG is considered to be an agent at the higher end of the moderate emetogenic risk category, analogous to carboplatin area under curve (AUC) ≥ 5 [28]. However, due to the absence of dedicated clinical trials evaluating antiemetic strategies for these agent, definitive evidence-based recommendations are currently lacking. In general, a two-drug regimen comprising a single doses of a 5-HT3 receptor antagonist and dexamethasone administered prior to SG, is recommended for most agents classified as moderate emetogenic category. In contrast, the NCCN guidelines, currently classify SG as a high emetogenic risk agent [29]. Also, The Delphi panel recommended a three-drug regimen including 5-HT3 antagonist, dexamethasone, and NK1 receptor antagonist from the first cycle, especially in patients at higher emetic risk [6]. In our study, the standard two-drug antiemetic regimen was effective for the majority of patients. However, 6% of patients required escalation to a three-drug regimen that included aprepitant, and 6% received olanzapine in the following cycles. Notably, no episodes of vomiting grade 3 or higher were observed, and only one patient experienced grade 3 nausea, a finding comparable to the 3% rate of grade 3 nausea reported in the ASCENT trial [3]. It is important to emphasize that nausea and vomiting associated with ADCs such as SG may present with delayed onset compared to traditional chemotherapy. In the ASCENT trial, the median time to onset was 24 days for vomiting and 8 days for nausea [3]. These unique temporal dynamics should be considered when designing antiemetic prophylaxis and patient education strategies.

The most clinically significant toxicities are those that interfere with the ability to maintain adequate treatment intensity. In our cohort, one in four patients (25%) required at least one dose reduction, primarily due to neutropenia. Adverse events leading to permanent treatment discontinuation were infrequent, with only two patients (3%) discontinuing therapy due to toxicity. These findings are consistent with the ASCENT trial, where 22% of patients required dose reductions and treatment discontinuation due to adverse events occurred in five patients [3]. Furthermore, according to the Delphi panel, dose reductions and delays, when applied in line with the characteristics SmPC, were not perceived to compromise treatment efficacy, supporting flexibility in clinical decision-making [6].

Adequate prophylaxis against hypersensitivity reactions is essential when administering SG. In the ASCENT trial, any-grade hypersensitivity events occurring within 24 h of dosing were reported in 34% of patients, with grade 3 or higher events occurring in 1.7% [3]. A pooled safety analysis reported hypersensitivity reactions of any grade and grade 3 or higher in 35% (369 patients) and 2% (17 patients), respectively [27]. In our cohort, 10% of patients did not receive acetaminophen at any point during treatment, and approximately 80% received H1 and H2 blockers as premedication. Despite this variation in premedication patterns, a hypersensitivity reaction grade 3 or higher was reported in only one patient. Nevertheless, adherence to current prophylactic guidelines remains crucial to minimizing the risk of infusion-related reactions.

The data from this multicenter cohort study provide important insights into premedication practices for sacituzumab govitecan treatment in TNBC patients. Consistent with current guidelines, the majority of patients received a standard regimen of acetaminophen, corticosteroids, and 5-HT3 antagonists prior to their SG infusions. The need for G-CSF, however, showed considerable variation between centers, with a high proportion of patients eventually requiring this intervention for either primary or secondary prophylaxis.

### Study limitations

This study has several limitations that warrant careful consideration when interpreting the findings. As an observational, multicenter analysis based on real-world data, it is inherently subject to variability in institutional practices and clinical judgment, which may influence the consistency and reproducibility of supportive care patterns observed. While the high rate of G-CSF use appears clinically plausible given the known toxicity profile of SG, this outcome should not be overinterpreted as confirmatory evidence in the absence of standardized protocols or controlled comparison. The retrospective nature of data collection may also introduce recall bias, and the relatively small sample size limits generalizability. Variability in treatment practices across multiple oncology centers, particularly regarding G-CSF administration and premedication strategies, could influence the findings. Data regarding antidiarrheal management were limited to atropine prophylaxis, as the use of agents such as loperamide was not systematically recorded. Additionally, the focus on short-term AEs without long-term follow-up data prevents evaluation of sustained outcomes such as overall survival. Data on potential de-escalation of premedication in subsequent cycles were not systematically collected, which may limit the interpretation of longitudinal supportive care practices. The inclusion of patients with diverse metastatic involvement and prior treatment histories adds heterogeneity, while the absence of genetic and biomarker analysis, such as UGT1A1 genotyping, limits the ability to identify patients at increased risk for toxicities. However, the Delphi panel did not recommend routine UGT1A1 testing, although closer monitoring was advocated in known *28/*28 homozygotes due to higher rates of severe hematologic and gastrointestinal toxicities [6].

### Clinical considerations

The findings from this multicenter study provide important insights into supportive care strategies during SG treatment in patients with TNBC. The observed heterogeneity in G-CSF use reflects the absence of unified national recommendations during the study period. At that time, decisions regarding prophylaxis were based on institutional protocols and clinical judgment. While some centers limited G-CSF use to reactive indications following neutropenic events, others implemented primary prophylaxis in selected high-risk patients. The recently updated SmPC (June 2025) formally endorses such an approach, offering a regulatory framework that aligns with several of the previously observed practices. This update, discussed in detail in Supplementary Materials 3, may support more standardized implementation of prophylaxis in future clinical settings. Several areas warrant further attention:

- G-CSF use: Early G-CSF initiation by the second cycle day 1 suggests significant myelosuppression, highlighting the need for standardized primary prophylaxis protocols to prevent treatment delays.
- Atropine for diarrhea management: Limited use of atropine despite its established role in managing cholinergic toxicity suggests the need for clearer recommendations for gastrointestinal prophylaxis.
- Biomarker-based risk stratification: The absence of UGT1A1 genotyping, despite its relevance in predicting severe neutropenia, reflects a gap in personalized supportive care.

## Conclusion

Premedication regimens for SG in patients with TNBC are generally uniform. The majority of patients received acetaminophen, histamine H1 and H2 receptor antagonists, corticosteroids, and 5-HT3 receptor antagonists as part of their premedication regimen. Adverse events linked with SG treatment are well documented, resulting in similar prophylactic measures for most patients. However, there was notable variability in the administration of G-CSF for both primary and secondary prophylaxis, with numerous patients requiring its initiation as early as the second treatment cycle. Furthermore, only a limited number of patients required atropine for secondary prophylaxis against diarrhea. Our findings, although limited by their retrospective nature, are broadly consistent with expert recommendations, which emphasize personalized G-CSF and antiemetic strategies, close monitoring for neutropenia in high-risk patients, and the need for further prospective evidence to refine supportive care in SG-treated TNBC [6].

## Supplementary Information

Below is the link to the electronic supplementary material.

ESM 1 (DOCX 21.4 KB)

## Data Availability

The datasets generated and analyzed during the current study are available from the corresponding author on reasonable request.

## Abbreviations

*AE*: Adverse event
*ALT/AST*: Aminotransferase/aspartate aminotransferase
BC: Breast cancer
*HER2*: Human epidermal growth factor receptor 2
*PARP*: Poly (ADP-ribose) polymerase
*5-HT3*: 5-hydroxytryptamine type 3 receptor
*H1*: Histamine type 1 receptor
*H2*: Histamine type 2 receptor
*ND*: No data
