# Supplementary material for: Strategies for premedication and G-CSF application in sacituzumab govitecan treatment of patients with triple-negative breast cancer: multicenter insights
Source: Support Care Cancer. 2025 Sep 28;33(10):889. doi: 10.1007/s00520-025-09918-4 (PMC12477073; doi:10.1007/s00520-025-09918-4)
Supplement: Supplementary file 1 — (DOCX 21.4 KB) [file 520_2025_9918_MOESM1_ESM.docx]

**Supplementary Materials 1: Reimbursement program criteria for sacituzumab govitecan in triple negative breast cancer treatment**

Sacituzumab govitecan monotherapy is indicated for the treatment of patients with metastatic or locally advanced unresectable triple-negative breast cancer (TNBC) in the second, third, or fourth line of treatment. Eligibility requires prior administration of at least two lines of systemic palliative therapy. For patients who have received perioperative treatment, at least one line of systemic palliative therapy is mandatory.

**Eligibility criteria:**

- Age ≥18 years.

- Histologically confirmed advanced breast cancer:

- Metastatic breast cancer (Stage IV), or

- Locally advanced breast cancer where radical local treatment (surgery or radiotherapy) is ineffective or unfeasible (Stage III).

- Histologically confirmed triple-negative breast cancer.

- Documented absence of steroid hormone receptor expression (ER and PR <1%).

- Documented absence of HER2 overexpression (IHC score of 0 or 1+) or HER2 gene amplification (negative ISH result).

- Measurable disease assessable according to RECIST criteria.

- ECOG performance status of 0-1.

- Pregnancy and breastfeeding exclusion.

- Absence of life-threatening visceral metastases.

- No clinically significant, uncontrolled comorbidities.

- Absence of symptomatic or progressing CNS metastases following local treatment (surgery or radiotherapy).

- Adequate organ function confirmed by laboratory tests to ensure treatment safety.

- In cases of concurrent malignancies, treatment eligibility must consider the prognosis related to the coexisting cancer.

All the above criteria must be met collectively.

**Criteria for treatment discontinuation:**

- Disease progression confirmed according to current RECIST criteria.

- Clinically significant deterioration of the patient's condition related to cancer without confirmed progression in imaging or physical examination.

- Toxicity requiring treatment discontinuation, as determined by the treating physician per the current Summary of Product Characteristics (SmPC).

- Decline in performance status:

- ECOG 2-4 for early-stage HER2-positive and TNBC treatment.

- ECOG 3-4 for metastatic HER2-negative or HER2-positive breast cancer treatment.

- Hypersensitivity to the drug, murine protein, or any excipient preventing treatment continuation.

- Significant quality of life deterioration, as assessed by the treating physician.

- Pregnancy or breastfeeding, unless the risk-benefit ratio justifies continuation following consultation with the National or Regional Consultant.

- Lack of cooperation or non-compliance with medical recommendations, particularly concerning scheduled assessments for treatment efficacy and safety.

*Abbreviations*: CNS, central nervous system; ECOG, Eastern Cooperative Oncology Group; ER, oestrogen receptor; HER2, human epidermal growth factor receptor 2; IHC, immunohistochemistry; ISH, in situ hybridisation; PR, progesterone receptor; RECIST, Response Evaluation Criteria in Solid Tumours; SmPC, Summary of Product Characteristics; TNBC, triple-negative breast cancer.

**Supplementary Materials 2: Dosing modifications for severe neutropenia as per drug product characteristics**

The SmPC outlines dose adjustments and the introduction of G-CSF based on the severity and duration of neutropenia:

1. Grade 4 neutropenia (lasting ≥7 days) or
2. Grade 3-4 febrile neutropenia, or
3. Grade 3-4 neutropenia causing a treatment delay of 2-3 weeks for recovery to ≤ Grade 1:
   - First occurrence: Initiate G-CSF as soon as clinically indicated.
   - Second occurrence: Reduce the dose of sacituzumab govitecan by 25% and administer G-CSF as clinically indicated.
   - Third occurrence: Reduce the dose by 50% and continue G-CSF administration as clinically indicated.
   - Fourth occurrence: Discontinue treatment with sacituzumab govitecan and administer G-CSF as clinically indicated.
4. Grade 3-4 neutropenia delaying treatment by more than 3 weeks for recovery to ≤ Grade 1:
   - Discontinue sacituzumab govitecan and administer G-CSF.

Monitoring and administration:

- Pre-treatment monitoring: Blood counts should be monitored before each treatment cycle.
- During treatment: G-CSF should be administered at the first sign of severe neutropenia as per the outlined guidelines.
- Duration: G-CSF is typically continued until neutrophil counts have recovered to an acceptable level for chemotherapy continuation.

Prophylaxis recommendations:

- Primary prophylaxis: G-CSF may be initiated in patients at high risk of neutropenic complications before the onset of neutropenia.
- Secondary prophylaxis: If neutropenia recurs despite dose reductions, G-CSF should be used to maintain adequate neutrophil counts.

*Abbreviations*: G-CSF, Granulocyte Colony-Stimulating Factor; SmPC, summary of product characteristics

**Supplementary Materials 3: Recommendations for G-CSF prophylaxis and neutropenia management based on the updated Summary of Product Characteristics (June 2025)**

Primary prophylaxis

Initiation of G-CSF at the start of treatment should be considered in patients with a heightened risk of developing neutropenic complications. Risk factors include age ≥65 years, previous episodes of neutropenia or febrile neutropenia, reduced bone marrow reserve due to prior therapies, comorbidities (e.g., renal or hepatic impairment), or impaired performance status. In such patients, G-CSF may help minimize the risk of severe neutropenia from the first cycle.

Secondary prophylaxis

In patients who experience clinically significant neutropenia or febrile neutropenia during earlier treatment cycles, G-CSF should be introduced in subsequent cycles to reduce the likelihood of recurrence and treatment delays. Its continued use may support adherence to planned dose intensity when neutropenic complications have already emerged.

Neutropenia-related treatment modifications

Dose adjustments and G-CSF use are advised according to the duration and clinical consequences of neutropenia:

- Grade 4 neutropenia lasting at least 7 days,
- Febrile neutropenia of grade 3 or 4, or
- Grade 3–4 neutropenia requiring 2–3 weeks for recovery to grade 1 or lower:

• First event: Initiate G-CSF without dose reduction.
• Second event: Reduce sacituzumab govitecan dose by 25% and continue G-CSF.
• Third event: Apply a 50% dose reduction and maintain G-CSF.
• Fourth event: Discontinue sacituzumab govitecan and proceed with G-CSF support.

If recovery from grade ≥3 neutropenia is delayed by more than 3 weeks, permanent discontinuation of sacituzumab govitecan is recommended, along with G-CSF administration.

Management of febrile neutropenia

In cases of fever (single measurement ≥38.3°C or sustained ≥38.0°C for over one hour) with neutropenia (ANC <1.0 × 10⁹/L), patients should be promptly evaluated and receive empirical broad-spectrum antibiotics. Hospital admission is advised for individuals with clinical instability, organ dysfunction, or other high-risk features. G-CSF use in this context follows institutional supportive care guidelines.

Monitoring recommendations
Full blood counts should be obtained prior to each treatment cycle and reassessed as necessary during therapy. G-CSF may be administered as daily filgrastim or as a long-acting formulation (e.g., pegfilgrastim or lipegfilgrastim), per institutional preference and clinical indications.

*Abbreviations*: ANC, absolute neutrophil count; G-CSF, granulocyte colony-stimulating factor; SmPC, summary of product characteristics.
